# Past, Present, and Future Shared Decision-making Behavior Among Patients With Eczema and Caregivers

**DOI:** 10.1001/jamadermatol.2022.2441

**Published:** 2022-07-06

**Authors:** Isabelle J. Thibau, Allison R. Loiselle, Emile Latour, Erin Foster, Wendy Smith Begolka

**Affiliations:** 1 National Eczema Association, Novato, California; 2 Biostatistics Shared Resource, Knight Cancer Institute, Oregon Health & Science University, Portland; 3 Center for Health & Healing, Oregon Health & Science University, Portland

## Abstract

**Importance:**

Engaging in shared decision-making (SDM) can help patients understand and choose treatments according to their values and has been shown to improve patient satisfaction and adherence with treatment. It is well suited for conditions like eczema for which several medically appropriate treatment options exist. However, little is known about the use and experience of SDM in eczema.

**Objective:**

To understand experiences, preferences, and expectations for SDM in eczema care settings from the patient and caregiver perspective.

**Design, Setting, and Participants:**

The National Eczema Association conducted an online survey in January 2021 among self-selected patients and caregivers. Inclusion criteria (US resident, patient with eczema or caregiver of a patient with eczema aged ≤17 years, respondent age ≥18 years) were met by 1313 of 1387 respondents (94.7%). Data analysis was performed from May 2021 to November 2021.

**Main Outcomes and Measures:**

Outcomes were past SDM (using a 9-item SDM questionnaire [SDMQ9]; score ranges transformed to 0-100), present SDM (Control Preferences Scale), and future SDM (self-reported confidence and motivation to engage in SDM) behavior.

**Results:**

Among the 1313 respondents included in the study, most were female (1046 of 1313 [79.7%]) adult (1086 of 1313 [82.7%]) patients with a mean (SD) patient Recap of Atopic Eczema score of 11.7 (7.2), mean (SD) patient age of 39.5 (22.2) years, and mean (SD) SDMQ9 score of 65.1 (27.4). For present SDM, 479 of 966 (49.6%) reported “I prefer to make the final decision after seriously considering my doctor’s opinion,” and 655 of 955 (69.4%) reported being very or extremely confident to engage in SDM in the future. Those who reported feeling “very well informed” about the causes of eczema had a 14.7-point higher (95% CI, 9.2-20.2; *P* < .001, multiple linear regression) SDMQ9 score than those “not adequately informed” and were 3.4 times more likely (95% CI, 2.1-5.7; *P* < .001, multiple logistic regression) to be confident to engage in future SDM. Respondents reported that they would be motivated to engage in SDM if the clinician initiated SDM, valued input from the patient, and acknowledged that patients are experts on their own bodies or if a treatment is no longer working.

**Conclusions and Relevance:**

Results of this survey study suggest that a majority of patients with eczema and caregivers prefer a large role in decision-making for their care and that clinicians can initiate and facilitate SDM to improve patient satisfaction with care.

## Key Points

**Question:**

What are the experiences, preferences, and motivators for shared decision-making (SDM) in patients with eczema?

**Findings:**

In this survey study of 1313 patients with eczema and caregivers, higher degree of involvement in SDM was significantly associated with higher consultation satisfaction, and self-reported knowledge about the causes of eczema was associated with past and future SDM. Control of disease was inversely associated with past SDM behavior; motivating factors for patients to engage in SDM included the clinician welcoming the patient’s input, acknowledgment that patients are experts on their bodies, and whether treatment is working.

**Meaning:**

Asking for patient perspectives and empowering patients and caregivers through education can encourage SDM and improve patient satisfaction with care.

## Teaser

This survey study characterizes the experiences, preferences, and expectations for shared decision-making in eczema care settings from the patient and caregiver perspective.

## Introduction

*Eczema* is a term that encompasses a group of conditions that cause the skin to become itchy and inflamed. National estimates for the prevalence of eczema indicate that 1 in 10 individuals will be affected by eczema at some point in their lifetime.^1^ Atopic dermatitis, the most common form of eczema, can have both pediatric and adult onset.^2,3,4^ After initial presentation, eczema can be continuous for many years, or it can show a relapsing-remitting nature.^3^ Disease severity, specific phenotypes of the disease, triggers for flares, and quality-of-life effects are extremely patient-specific, necessitating an individualized treatment approach. The patient journey to achieving desired care experience and outcomes can additionally be challenging, time consuming, and negatively affect overall burden of disease.^5,6,7^

Shared decision-making (SDM) is a practice in which a health care practitioner (HCP) works with a patient or caregiver to incorporate their unique preferences and values into decisions about medically appropriate treatment options.^8^ Eczema is particularly well suited for SDM owing to its clinical and patient-reported heterogeneity that may allow for varying treatment approaches that can be informed by preference-sensitive decision-making.^9^ However, little is known about experiences with SDM among patients with eczema and caregivers in a clinical setting in the US.

This study’s objective is to investigate how patients with eczema and caregivers of pediatric patients with eczema have experienced SDM in the past, what their current preferences are to engage in SDM, and their level of confidence and motivation to engage in SDM in the future. As the therapeutic landscape for eczema is evolving, this study’s results can inform HCPs about the readiness of patients with eczema to engage in SDM with the goal of facilitating positive health care experiences and ultimately better health outcomes.

## Methods

The study included US (including US territories) adult (age ≥18 years) residents with a self-reported diagnosis of eczema (eczema type not asked or defined) or primary caregivers for pediatric patients with eczema. Survey availability was communicated to all National Eczema Association (NEA) members via the NEA website, email, social media, and the NEA EczemaWise app and distributed more broadly through Facebook and Google ads.

Prior to completing the 64-question online survey (eAppendix in the Supplement), respondents provided electronic informed consent, and those who fully completed the survey were eligible for 1 of 20 $50 Amazon.com gift cards. Survey responses were not linked in any way to the gift cards. This study was identified as exempt by the Western Institutional Review Board Copernicus Group. All data were anonymized for analysis and treated confidentially.

### SDMQ9—Past

The 9-item SDM questionnaire (SDMQ9) rates the extent to which patients are involved in the process of SDM.^10^ Responses to the 9 questions can be answered on a 6-point Likert scale ranging from 0 (“completely disagree”) to 5 (“completely agree”). These scores were summed and linearly transformed to create an intuitive score range of 0 to 100, with a higher score indicating higher level of involvement with SDM.^11^ This variable was used to explain past SDM experience, specifically the most recent eczema consultation.

### CPS—Present

**Figure 1.**
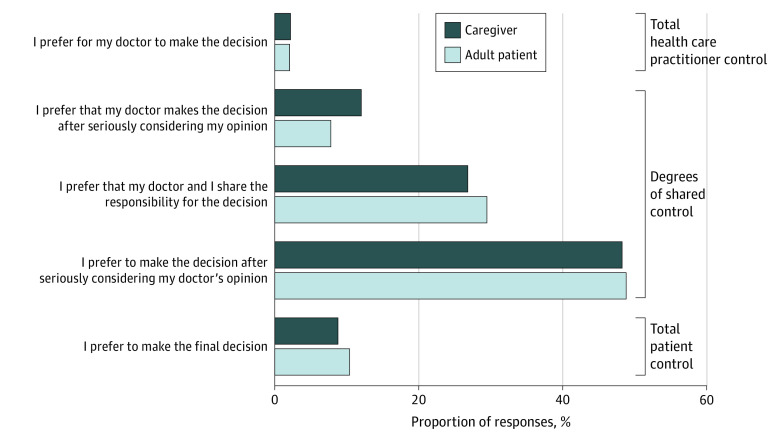
Control Preferences Scale (CPS) Question: “Please choose the statement that most accurately reflects your preferences regarding your role in making a decision regarding the treatment of your/your child's eczema problems.”

The Control Preferences Scale (CPS) asks how much control a patient/caregiver wants when it comes to making a decision about their treatment.^12^ All answer options are shown in Figure 1. This variable was used to explain present preferences for engaging in SDM.

### SDM Confidence—Future

Respondents were asked: “How confident do you feel about engaging in shared decision-making with your primary eczema healthcare provider?” (extremely confident, very confident, moderately confident, slightly confident, not confident at all). This variable was used to explain confidence to engage in future SDM.

### Explanatory Variables

Four final variables were included for modeling. (1) Respondents were asked about their knowledge about the causes of eczema (Question: “How well informed do you feel you are about the underlying causes of eczema?” (grouped into very well informed, adequately informed, not adequately informed). (2) The Recap of Atopic Eczema (RECAP) is a 7-item questionnaire designed to measure eczema control over the past week, with a lower score signifying more control.^13^ (3) Familiarity with SDM was assessed by asking if the respondent was aware of SDM before taking this survey. (4) Patient and caregiver feelings about the value of their opinions were also ascertained with the question: “I feel that I have ‘something to add’ to a discussion with a healthcare provider about my/my child’s condition” using a 5-point Likert scale.

### Statistical Analysis

Respondent demographics were described using means and SDs for continuous variables and counts and percentages for categorical variables. Past SDM experience was modeled using multiple linear regression. Variables were screened for inclusion using the purposeful selection modeling strategy.^14^ The final explanatory variables included were self-reported knowledge about the causes of eczema, RECAP score, familiarity with SDM, and a feeling of having valued opinions. These variables were also used to model future SDM confidence (binary: “very or extremely” confident or not) using multiple logistic regression. In both models, adult patients and caregivers were included together because the respondent was the person interacting with the HCP. For present preferences for SDM, a χ^2^ test for comparison of proportions was used on adult vs caregiver CPS responses to determine if parents felt a different sense of control over a child’s care than adults did for their own care.

A 1-way analysis of variance test was performed to determine if SDMQ9 score differed by the levels of satisfaction with that same visit. Participants were asked: “How would you rate your satisfaction with your overall care experience for this specific consultation?” (very dissatisfied, dissatisfied, unsure, satisfied, very satisfied). A post hoc Tukey test was completed to compare pairwise differences in mean SDMQ9 score between the groups. Significance level was adjusted for 10 comparisons.

Analysis was performed using R, version 4.1.0 (R Foundation for Statistical Computing). *P* values less than .05 were considered statistically significant unless otherwise indicated. There were no imputations performed for missing data; some participants were excluded from some analyses owing to missing data, which is indicated where applicable.

### Motivation

Participants were asked: “What would motivate you to engage in shared decision-making with your/your child’s healthcare provider?” Data were extracted using Dedoose, version 8 software (SocioCultural Research Consultants, LLC) for qualitative analysis by 2 reviewers (A.R.L. and E.F.). Thematic analysis was conducted, for which the final themes and subcodes for the question can be found in Table 1. Excerpts were reviewed by coders to determine interrater reliability, and discrepancies were resolved through discussion and consensus (with a third party, I.J.T.). A combination of deductive and inductive coding was used. The final interrater reliability was 97.4%.

**Table 1.** Major Themes and Subcodes for Qualitative Analysis of Motivation to Engage in SDM^a^

Major theme	Subcode	Major theme, No. (%)
Already motivated (n = 731)	Already motivated	102 (13.9)
Aspects about the patient (n = 224)	Expert on own body	77 (34.4)
	Concern for health	73 (32.6)
	Health literacy	31 (13.8)
	Patient has done research on eczema	27 (12.0)
	Communication skills	16 (7.1)
Aspects about the HCP (n = 213)	HCP values input	74 (34.7)
	HCP initiates SDM	61 (28.6)
	Trust in HCP	30 (14.1)
	HCP expresses genuine concern	23 (10.8)
	HCP discusses whole well-being	13 (6.1)
	Accepts alternative treatments	10 (4.7)
	HCP consults about triggers	2 (0.9)
Aspects about the visit (n = 122)	Treatment is working/not working	77 (63.1)
	Time with HCP	26 (21.3)
	Safe space	10 (8.2)
	Office environment	7 (5.7)
	In-person visit	1 (0.8)
	Proximity to clinic	1 (0.8)
Aspects about guidance (n = 71)	Multiple recommendations	46 (64.8)
	Preparation (having information on treatments beforehand)	22 (31.0)
	One clear recommendation	2 (2.8)
	Handout to structure discussion	1 (1.4)

Abbreviations: HCP, health care practitioner; SDM, shared decision-making.

^a^ Question: “What would motivate you to engage in shared decision-making with your/your child’s healthcare provider?”

## Results

There were 1387 complete survey respondents (1387 of 2836; complete response rate of 48.9%). Unfinished survey responses (74) were removed from analysis. Table 2 shows the characteristics of the included study population (n = 1313). The majority of patients identified as female (1046 of 1313 [79.7%]), with a mean (SD) age of 46.2 (17.9) years for adult patients and 7.4 (5.0) years for pediatric patients.

**Table 2.** Characteristics of the Study Population

Characteristic	All	Adult	Caregiver
Total respondents, No. (%)	1313 (100.0)	1086 (82.7)	227 (17.3)
Patient age, mean (SD), y	39.5 (22.0)	46.2 (17.9)	7.4 (5.0)
Age at diagnosis, mean (SD), y	17.2 (21.8)	20.8 (22.5)	1.5 (2.5)
Time since diagnosis, mean (SD), y	22.5 (20.4)	26.3 (20.7)	5.9 (4.7)
Gender of patient, No. (%)			
Female	1046 (79.7)	859 (79.1)	187 (82.4)
Male	249 (18.9)	211 (19.4)	38 (16.7)
Nonbinary	13 (1.0)	13 (1.2)	0
Other	5 (0.4)	3 (0.3)	2 (0.9)
Race of respondent, No. (%)			
Asian or Asian American	125 (9.5)	103 (9.5)	22 (9.8)
Black or African American	167 (12.7)	125 (11.5)	42 (18.5)
Multiracial	65 (5.0)	54 (5.0)	11 (4.8)
Native American or Alaska Native	15 (1.1)	11 (1.0)	4 (1.8)
Native Hawaiian or Pacific Islander	3 (0.2)	2 (0.2)	1 (0.4)
White	873 (66.5)	741 (68.2)	132 (58.1)
Some other race or ethnicity	35 (2.7)	27 (2.5)	8 (3.5)
Do not know/prefer not to say	30 (2.3)	23 (2.1)	7 (3.1)
Respondent ethnicity			
Hispanic/Latinx (% yes)	134 (10.2)	104 (9.6)	30 (13.2)
Education			
High school, technical postsecondary, some or no high school	158 (15.7)	137 (16.3)	21 (12.5)
Completed some college	215 (21.4)	179 (21.3)	36 (21.4)
4-y College degree	335 (33.3)	289 (34.4)	46 (27.4)
Master’s degree/doctorate	299 (29.7)	234 (27.9)	65 (38.7)
Income, $			
≤24 999	172 (17.1)	153 (18.2)	19 (11.3)
25 000 to 49 999	194 (19.3)	165 (19.7)	29 (17.3)
50 000 to 74 999	176 (17.5)	152 (18.1)	24 (14.3)
75 999 to 99 999	133 (13.2)	115 (13.7)	18 (10.7)
100 000 to 124 999	118 (11.7)	92 (11.0)	26 (15.5)
125 000 to 149 999	72 (7.1)	58 (6.9)	14 (8.3)
≥150 000	142 (14.1)	104 (12.4)	38 (22.6)

Most respondents (708 of 957 [74.0%]) were satisfied or very satisfied with their most recent consultation with an HCP. The mean (SD) total SDMQ9 score for this visit was 65.1 (27.4) (scale, 0-100). There were no significant differences in SDMQ9 score between people of different races or genders, but those who identified as non-Hispanic had higher SDMQ9 scores (engaged in more SDM in their past consultation) than those who identified as Hispanic (mean score: 57.1 for Hispanic respondents vs 65.9 for Non-Hispanic respondents; *t* test, *P* = .005). Additionally, SDMQ9 score did not differ significantly between lower and higher education levels (mean score: 64.6 for higher education vs 56.3 for lower education; *t* test, *P* = .16).

For the SDM past experience model, 335 respondents were excluded because of lack of responses or because they reported not currently seeing any HCP. Therefore, 978 responses were included in the model. Age, education level, and income were not significant in the multivariable model and thus were excluded from the final model. Those who felt “very well informed” about the causes of eczema had, on average, a 14.7-point higher SDMQ9 score (95% CI, 9.2-20.2 points higher; *P* < .001; Table 3). For every 1-point increase in RECAP score, SDMQ9 score decreased by an average 0.5 points (95% CI, 0.3-0.8 points decrease; *P* < .001). Respondents familiar with the term *SDM* before the survey had higher SDMQ9 score, on average, than those not familiar (6.0; 95% CI, 1.6-10.4 points higher; *P* = .007). The SDMQ9 score was 10.9 points higher (95% CI, 3.1-18.8; *P* = .006) for those who felt their opinions were valued compared with those who did not.

**Table 3.** Linear Regression Model With Past SDM (SDMQ9 Score) as Outcome and Logistic Regression Model With Future SDM (Confidence) as Outcome

Outcome	Linear regression for past SDM		Logistic regression for future SDM	
	Adjusted estimate	Adjusted *P* value	Adjusted odds ratio	Adjusted *P* value
Self-reported knowledge of causes of eczema				
Not adequately informed (reference)	NA	NA	NA	NA
Adequately informed	11.5 (6.4 to 16.6)	<.001	1.8 (1.1 to 2.7)	.01
Very well informed	14.7 (9.2 to 20.2)	<.001	3.4 (2.1 to 5.7)	<.001
RECAP score	−0.5 (−0.8 to −0.3)	<.001	1.0 (0.9 to 1.0)	.02
Familiarity with SDM				
No (reference)	NA	NA	NA	NA
Yes	6.0 (1.6 to 10.4)	.007	2.4 (1.6 to 3.5)	<.001
Feeling of valued opinions with HCP				
Disagree (reference)	NA	NA	NA	NA
Neutral	2.8 (−6.5 to 12.0)	.56	0.6 (0.3 to 1.3)	.22
Agree	10.9 (3.1 to 18.8)	.006	2.4 (1.2 to 4.5)	.01

Abbreviations: HCP, health care practitioner; NA, not applicable; RECAP, Recap for Atopic Eczema; SDM, shared decision-making; SDMQ9, 9-item SDM questionnaire.

For present SDM (CPS), 479 of 966 (49.6%) reported “I prefer to make the final decision after seriously considering my doctor’s opinion” with the next most popular response (284 of 966 [29.4%]) being “I prefer that my doctor and I share responsibility for the decision.” Preferences for control were similar between adult patients and caregivers of pediatric patients with eczema (Figure 1), and there was no difference in preferences by gender, age, or education level of the respondent.

The majority (655 of 944 [69.4%]) of respondents felt very or extremely confident to engage in SDM in the future. There was a significant difference between males (135 of 174 [77.6%]) and females (516 of 758 [68.1%]) being confident to engage (χ^2^ test; *P* = .01). In the multivariable model, age, education level, and income were not significant. Those who reported feeling “very well informed” about the causes of eczema were 3.4 times more likely (95% CI, 2.1-5.7 times; *P* < .001, Table 3) to be “very or extremely” confident to engage in SDM in the future compared with those who did not feel adequately informed. For every 1-point increase in RECAP score, the odds of feeling confident to engage in SDM in the future changed by 1.0 times (95% CI, 0.9-1.0 times; *P* = .02). Familiarity with SDM before the survey and feeling of valued opinions were both associated with higher odds of feeling confident to engage in SDM in the future (odds ratio, 2.4; 95% CI, 1.6-3.5; *P* < .001 and odds ratio, 2.4; 95% CI, 1.2-4.5; *P* = .01, respectively).

**Figure 2.**
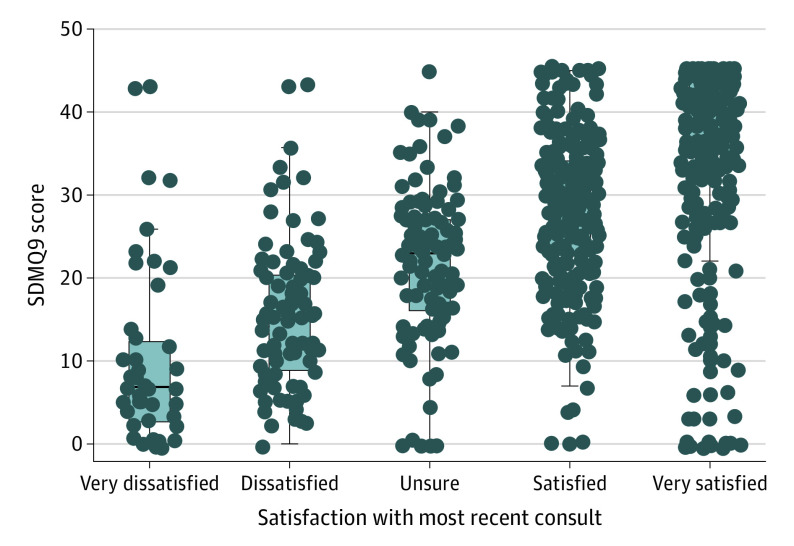
SDMQ9 Score Compared With Consult Satisfaction for a Recent Eczema Consultation Boxes and lines demonstrate the median and IQR of 9-item shared decision-making questionnaire (SDMQ9) scores, while whiskers mark the minimum (quartile 1 − 1.5 × IQR) and maximum (quartile 3 + 1.5 × IQR). Dots indicate individual SDMQ9 scores.

The analysis of variance test for SDMQ9 score and consult satisfaction for the most recent visit revealed that mean SDMQ9 score was not the same for different levels of consult satisfaction (*P* < .001; mean scores: very dissatisfied, 20.9; dissatisfied, 33.8; unsure, 48.3; satisfied, 62.5; very satisfied, 82.9). A post hoc Tukey test showed that the pairwise differences in mean SDMQ9 score between all groups were significant, with a higher mean SDMQ9 score with each increasing level of satisfaction (Figure 2).

Table 1 shows thematic codes and subcodes for the question, “What would motivate you to engage in SDM?” There were 731 responses, and the major themes included aspects about the patient, the HCP, the visit, and guidance. While not a response the question was directly eliciting, 13.9% (102 of 731) of all respondents reported already being motivated to engage in SDM. For those who did not mention being already motivated, the primary patient-related aspect (n = 224) was a perception of being an “expert on their own body” (77 of 224 [34.4%]). The most important HCP-related motivators (n = 213) were the HCP valuing input from the patient (74 of 213 [34.7%]) and the HCP initiating SDM (61 of 213 [28.6%]). For aspects about the visit (n = 122), 77 of 122 (63.1%) would be motivated to engage in SDM if they felt their treatment was not working. Finally, regarding guidance (n = 71), respondents preferred having multiple recommendations for treatments (46 of 71 [64.8%]).

## Discussion

In this study, we show that many adult patients with eczema and caregivers of children with eczema have engaged to some extent in SDM in the past, currently prefer to have a large amount of control in decision-making for their care, and are confident to do so in the future. Specifically, patients and caregivers who feel they are informed about the causes of eczema and who have better-controlled eczema are more likely to engage in and feel confident about SDM. Engaging to a greater degree in SDM is associated with an increase in consult satisfaction, and patients and caregivers are motivated to engage in SDM when they have an HCP who initiates a discussion and values their opinion as the experts on their own body, or if they feel their treatment is no longer working.

Patients and HCPs can engage in SDM when considering a decision with multiple medically appropriate treatment options and for which the best option may rely, in part, on an individual’s preferences, unique lived burden of disease, and previous treatment experiences. While there is a paucity of research on eczema outcomes after SDM, there is evidence from other chronic diseases to suggest it could be useful to improve patient outcomes. In a survey study of more than 63 000 patients with multiple diseases in the US, use of a weighted SDM score found an association between poor SDM and poor patient-reported outcomes and quality-of-life indicators.^15^ In dermatology specifically, a recent meta-analysis by Morrison et al^16^ of 29 SDM articles found consistent support of the value of SDM for patients and caregivers and noted that HCPs who implemented SDM found improved patient satisfaction. This is echoed in the current study, as there was a positive linear association between the extent to which SDM occurred and how satisfied patients were with their most recent consultation (Figure 2). Patient satisfaction has also been associated with improved treatment adherence and therefore disease outcomes.^17^

In this study, the majority of patients with eczema and caregivers have practiced some degree of SDM in a past consultation, whether or not they knew before the survey what that practice was called. We found an average SDMQ9 score of 65.1 (a higher score means that more SDM occurred; range, 0-100), which was slightly higher than the score of 55 found in a Dutch patient population with atopic dermatitis and psoriasis.^9^ This could, in part, be explained by SDM awareness and cultural differences. Indeed, only 52% of this Dutch cohort were aware of the term *SDM* before the survey, while 76.1% of the current study cohort were aware. In the current study, the majority (49.6%) of patients wanted to make the final decision after seriously considering the HCP’s opinion, while that number was lower (30%) in the Dutch cohort.

Interestingly, the Dutch study^9^ also found discordance in the perception of how much SDM occurred, with HCPs reporting a score of 82 compared with the patients’ score of 55. In the current study, 69.4% of respondents felt very or extremely confident to engage in SDM in the future. Male respondents were more likely than female respondents to be confident, which is in line with previous research on gender differences in confidence.^18^ Discordance between the patient and HCP perspective in atopic dermatitis related to assessment of disease severity has been previously reported.^19^ While this study did not address the HCP perspective on SDM in eczema, these data suggest that there could be a gap in the patient and HCP perception of how much SDM has occurred, highlighting an important area for further investigation.

According to research by Elwyn et al,^20^ there are 3 conditions for patients that must be met for SDM to be used in normal clinical practice: (1) access to evidence-based information about different treatments, (2) guidance on how to make an informed decision about those treatment options, and (3) a supportive clinical environment that encourages and values patient engagement. In the current study, in both the past SDM experience and future confidence to engage models, self-reported knowledge stood out as a primary contributor, which was in line with the first 2 proposed conditions. Previous research has shown that education for patients with eczema led to a reduction in skin symptoms and better coping with the disease^21^ and that patient activation in general is associated with improved treatment adherence, health outcomes, and the care experience.^22,23^ Many tools have been suggested to educate patients with eczema and provide them with guidance on treatment options, but few have been evaluated in practice.^24,25,26^

The final condition addresses a supportive environment for patients to engage in SDM. There is a somewhat paradoxical relationship in that patients may become more confident if they are more informed about eczema treatments, but they may not become more informed unless they are confident enough to ask questions in the first place. In this study, we found that many patients with eczema and caregivers reported they would be motivated to engage in SDM if their HCP initiated the discussion, if they felt their opinions were valued, and if they were recognized as the expert on their or their children’s bodies. Patients and caregivers may become more equipped to initiate conversations about treatment options as structures for engaging in SDM become more established in clinical practice. Health care practitioners can actively initiate SDM conversations as well as evaluate their current approach for introducing SDM in the care setting at individual visits and over time as treatment plans are refined or revised.

### Limitations

A limitation of this study was that survey respondents were largely part of NEA’s community and may have a higher level of knowledge of eczema and/or a different level of engagement with their HCP. It is possible that patients who are proactive in their care and are involved with organizations like NEA would be more informed about eczema and have the confidence to discuss treatments with their HCP. This study was also limited by its cross-sectional nature. However, the strength of the results demonstrated here represent an “upper limit” in knowledge of and capacity for SDM from a large, demographically and clinically diverse cohort of patients with eczema and caregivers from across the US. The general population with eczema could benefit even more from structured information on eczema and treatments, initiation of SDM from clinicians, and SDM guidance tools in a supportive clinical environment.

## Conclusions

In this survey study, higher SDMQ9 score was associated with greater satisfaction with that same consultation. Self-reported knowledge about the causes of eczema was associated with both past SDM and confidence to engage in SDM in the future. This suggests that HCPs have a unique opportunity to facilitate SDM in the care setting and empower patients and caregivers by embedding education into the patient interaction. Acknowledgment from the clinician that the patient and caregiver perspective is valuable may also potentially increase SDM, which can increase satisfaction with the visit, ultimately leading to increased adherence and better disease control.

## Notes

Supplement. **eAppendix.** National Eczema Association Shared Decision Making Survey
