# Supplementary material for: Past, Present, and Future Shared Decision-making Behavior Among Patients With Eczema and Caregivers
Source: JAMA Dermatol. 2022 Jul 6;158(8):912–8. doi: 10.1001/jamadermatol.2022.2441 (PMC9260637; doi:10.1001/jamadermatol.2022.2441)
Supplement: Supplement. — eAppendix. National Eczema Association Shared Decision Making Survey [file jamadermatol-e222441-s001.pdf]

# Supplemental Online Content

Thibau IJ, Loiselle AR, Latour E, Foster E, Smith Begolka W. Past, present, and future shared decision-making behavior among patients with eczema and caregivers. *JAMA Dermatol*. Published online July 6, 2022. doi:10.1001/jamadermatol.2022.2441

**eAppendix.** National Eczema Association Shared Decision Making Survey

This supplemental material has been provided by the authors to give readers additional information about their work.

© 2022 Thibau IJ et al. *JAMA Dermatology.*

**eAppendix. National Eczema Association Shared Decision Making Survey**

Opening Screen: Introduction

Welcome! Please read about the survey before you participate.

**This survey takes about 15-20 minutes**.

**Survey Topic**

The National Eczema Association (NEA) is committed to understanding all aspects of the experience of living with eczema, both for affected individuals and their families. **With the following survey, we are asking specifically about how you, as an eczema patient or caregiver of an eczema patient, work with your healthcare provider to gain a better understanding of the condition and treatment options.** This process is known as **shared decision making** and is particularly suitable for eczema, a variable condition for which many treatment options may exist.

**[INFORMED CONSENT OBTAINED HERE]**

—page break—

The following questions will determine if you are eligible to participate.

—page break—

1. Do you reside in the U.S. or its territories?
    a. Yes
    b. No (ineligible to participate)

2. Are you 18 years of age or older?
    a. Yes
    b. No (ineligible to participate)

—page break—

Demographics

3. CONNECTION TO ECZEMA Please indicate your **primary connection to eczema**. If you have more than one connection to eczema, please choose one that you will focus on for this survey.
    a. I am an adult (18 years or older) with eczema (adult branch)

© 2022 Thibau IJ et al. *JAMA Dermatology.*

    b. I am a parent/primary caregiver for a child aged 0-17 who has eczema (caregiver branch)
    c. Other (neither adult with eczema nor parent/caregiver of a minor) (screened out)

—page break—

4. IF CAREGIVER, AGE What is **your child's** age?
    a. Select from drop down: Younger than 1 year, … 17 years old, 18 years or older. (If 18+, screened out)
5. AGE What is your age?
    a. Select from drop down: 18 years old, 19 years old, … 90 years or older
6. GENDER What is your gender?
    a. Male
    b. Female
    c. Non-binary
    d. Other
7. RACE What is your race?
    a. White
    b. Black or African American
    c. Asian or Asian American
    d. Native American or Alaskan Native
    e. Native Hawaiian or Pacific Islander
    f. Multiracial
    g. Some other race or ethnicity
    h. I don't know/prefer not to answer
8. HISPANIC ETHNICITY Are you of Hispanic or Latino origin?
    a. Yes
    b. No

—page break—

## Eczema Condition

9. AGE OF DIAGNOSIS At what age were you/was your child first diagnosed with eczema?
    a. Select from drop down: Not sure, Less than 1 month, 1 month, 2 months, … 1 year, 2 years, ...90+ years.
10. IF NOT SURE Were you/Was your child first diagnosed at …..
    a. Under 2 years of age
    b. 2 to 5 years

© 2022 Thibau IJ et al. *JAMA Dermatology.*

c. 6 to 9 years
d. 10 to 17 years
e. 18 years or older
f. Not sure

—page break—

11. TRACKING Do you track your/your child's disease? Tracking may include keeping a journal or spreadsheet of symptoms and/or triggers, or keeping notes in a notebook.
    a. Yes
    b. No
12. SELF-ASSESSED DISEASE KNOWLEDGE How well informed do you feel you are about the underlying causes of eczema?
    a. Very well informed
    b. Adequately informed
    c. Not adequately informed

—page break—

ECZEMA CONTROL VIA SEVERITY AND QOL SERIES The questions below provide a snapshot of how your/your child's eczema has been over the last week from your point of view.

13. RECAP 1. Over the last week, **how has your/your child's eczema been**?
    a. Very good
    b. Good
    c. Ok
    d. Bad
    e. Very Bad
14. RECAP 2. Over the last week, on how many days has your/your child's **skin been itchy** because of your/their eczema?
    a. No days
    b. 1-2 days
    c. 3-4 days
    d. 5-6 days
    e. Every day
15. RECAP 3. Over the last week, on how many days has your **skin been *intensely* itchy** because of your eczema? / on how many days do you think your child's **skin has been *intensely* itchy** because of their eczema?
    a. No days
    b. 1-2 days

© 2022 Thibau IJ et al. *JAMA Dermatology.*

    c. 3-4 days
    d. 5-6 days
    e. Every day
16. RECAP 4. Over the last week, how much has your **sleep been disturbed** because of your eczema? / how much do you think your child's **sleep has been disturbed** because of their eczema?
    a. Not at all
    b. A little bit
    c. Quite a bit
    d. A huge amount
    e. Completely
17. RECAP 5. Over the last week, how much has your/your child's eczema been **getting in the way of day to day activities**?
    a. Not at all
    b. A little bit
    c. Quite a bit
    d. A huge amount
    e. Completely
18. RECAP 6. Over the last week, on how many days has your eczema **affected how you have been feeling**? / on how many days do you think your child's eczema **affected how they have been feeling**?
    a. No days
    b. 1-2 days
    c. 3-4 days
    d. 5-6 days
    e. Every day
19. RECAP 7. Over the last week, **how acceptable** has your/your child's eczema been to you?
    a. Completely acceptable
    b. Mostly acceptable
    c. Quite acceptable
    d. Not very acceptable
    e. Not at all acceptable

—page break—

Healthcare Provider/SDM Experience

© 2022 Thibau IJ et al. *JAMA Dermatology.*

20. NUMBER OF HCP How many providers do you see for your/your child's eczema care?
    a. 0 (I do not see any providers for my eczema care) (skip to Q57)
    b. 1
    c. 2
    d. 3
    e. 4
    f. 5
    g. 6
    h. 7
    i. 8
    j. 9
    k. 10+

—page break—

The following questions are about your/your child's **primary eczema healthcare provider.** This is the person you mainly see about your/your child's eczema. If you work closely with two or more healthcare providers, choose the person you *mainly* interact with to make decisions about your/your child's care.

In this section, we ask for various characteristics about this person to better understand their relationship with you and to collect information on factors that may be important in a shared decision-making experience.

21. CLIN SPECIALTY What type of medical professional is your/your child's primary eczema healthcare provider?
    a. General or Family Physician
    b. Dermatologist
    c. Allergist
    d. Physician Assistant/Nurse Practitioner
    e. Pediatrician (for child only)
    f. Other (please specify: _________ )
22. TIME WITH CLIN How long have you been seeing this particular healthcare provider? *Please indicate a number for both months and years.*
    a. __ months __ years
23. CLIN GENDER Does this healthcare provider have the same gender as you, as far as you can tell?
    a. Yes
    b. No
    c. Don't know

© 2022 Thibau IJ et al. *JAMA Dermatology.*

24. CLIN RACE Does this healthcare provider have the same race as you, as far as you can tell?
    a. Yes
    b. No
    c. Don't know
25. CLIN ETHNICITY Does this healthcare provider have the same ethnicity as you, as far as you can tell?
    a. Yes
    b. No
    c. Don't know
26. CLIN CULTURAL SENSITIVITY Does this healthcare provider express sensitivity to your culture? *Culture may refer to your particular social group, background, beliefs and way of life. Sensitivity may include knowledge, awareness and acceptance.*
    a. Every time
    b. Almost every time
    c. Sometimes
    d. Almost never
    e. Never

—page break—

27. JOURNEY How did you find your/your child's current primary eczema healthcare provider (the person you identified on the last page)?
    *Please describe what that journey looked like, such as how long the process was, how you felt, why you ended up with this person, etc.* [open text box]

—page break—

SDMQ9 SECTION Now, think back to a **recent eczema-related consultation** involving this same **primary eczema healthcare provider** that you identified earlier. For these questions, you should focus on a consultation where a decision needed to be made.
*This decision could be in regards to taking new therapy, continuing existing therapy, stopping a therapy you had been taking, making changes to your lifestyle or other aspects of your overall health that impact eczema, other management aspects, or a need to see a specialist, for example.*

28. TELEMED Was this a telemedicine visit?
    a. Yes—phone only
    b. Yes—phone and/or video
    c. No
29. LENGTH How long ago was this encounter?

© 2022 Thibau IJ et al. *JAMA Dermatology.*

    a. __ months (and years?) ago

30. (SDMQ9) Please indicate which health complaint/problem the consultation was about. If this was a routine or preventative visit, please indicate that. (write-in)
31. (SDMQ9) Please indicate what decision(s) were made. *This decision could be in regards to taking new therapy, continuing existing therapy, stopping a therapy you had been taking, making changes to your lifestyle or other aspects of your overall health that impact eczema, other management aspects, or a need to see a specialist, for example.* (write-in)

—page break—

(SDMQ9) Nine statements related to the decision-making in your consultation (that you identified on the previous page) are listed below. For each statement please indicate how much you agree or disagree.
[completely disagree, strongly disagree, somewhat disagree, somewhat agree, strongly agree, completely agree]

32. (SDMQ9) My healthcare provider made clear that a decision needs to be made.
33. (SDMQ9) My healthcare provider wanted to know exactly how I want to be involved in making the decision.
34. (SDMQ9) My healthcare provider told me that there are different options for treating my medical condition.
35. (SDMQ9) My healthcare provider precisely explained the advantages and disadvantages of the treatment options.
36. (SDMQ9) My healthcare provider helped me understand all the information.
37. (SDMQ9) My healthcare provider asked me which treatment option I prefer.
38. (SDMQ9) My healthcare provider and I thoroughly weighed the different treatment options.
39. (SDMQ9) My healthcare provider and I selected a treatment option together.
40. (SDMQ9) My healthcare provider and I reached an agreement on how to proceed.

—page break—

41. [CAREGIVER ONLY] CT SURVEY CHILD ENGAGEMENT. How engaged was your child in the decision making **for this particular decision**?
    a. Not at all engaged
    b. Engaged to some extent
    c. Engaged to a moderate extent
    d. Engaged to a great extent
    e. Engaged to a very great extent

© 2022 Thibau IJ et al. *JAMA Dermatology.*

42. [CAREGIVER ONLY] CT SURVEY CHILD ENGAGEMENT. How engaged is your child in the decision making regarding their eczema **in general**?
    a. Not at all engaged
    b. Engaged to some extent
    c. Engaged to a moderate extent
    d. Engaged to a great extent
    e. Engaged to a very great extent

—page break—

43. (CONTROL PREFERENCES SCALE-CPS) Please choose the statement that most accurately reflects your preferences regarding your role in making a decision regarding the treatment of your/your child's eczema problems:
    a. I prefer to make the final decision
    b. I prefer to make the final decision after seriously considering my doctor's opinion
    c. I prefer that my doctor and I share responsibility for the decision
    d. I prefer that my doctor makes the decision after he/she seriously considers my opinion
    e. I prefer my doctor to make the decision

—page break—

Again, think back to this same eczema-related consultation involving the primary eczema healthcare provider you identified on the previous pages**.**

44. SATISFACTION WITH CARE EXPERIENCE   How would you rate your satisfaction with your overall care experience for this specific consultation?
    a. Very dissatisfied
    b. Dissatisfied
    c. Unsure
    d. Satisfied
    e. Very satisfied
45. COMPARE WITH PREVIOUS VISITS How does the care experience for this consultation compare with previous visits with this *same healthcare provider*?
    a. This care experience was better than previous visits
    b. This care experience was about the same as previous visits
    c. This care experience was worse than previous visits
    d. N/A—This is my first care experience with this healthcare provider
46. COMPARE WITH OTHER HCP How does the care experience for this consultation compare with your care experience with *other eczema healthcare providers*?

© 2022 Thibau IJ et al. *JAMA Dermatology.*

a. This care experience was better than my experience with other eczema healthcare providers
b. This care experience was about the same as my experience with other eczema healthcare providers
c. This care experience was worse than my experience with other eczema healthcare providers
d. N/A—I don't see other healthcare providers for my/my child's eczema

——section break——

SDM Awareness and Factors

47. FAMILIAR Before taking this survey, were you familiar with the term "shared decision-making?"
    a. Yes
    b. No

—page break—

While there is no one definition for shared decision-making, it is generally considered a process by which both the patient (or caregiver of a patient) and the healthcare provider discuss the patient's experience with their condition and work together to identify appropriate treatment options.

48. CONFIDENCE How confident do you feel about engaging in shared decision-making with the primary eczema healthcare provider identified from the previous pages?
    a. Extremely confident
    b. Very confident
    c. Moderately confident
    d. Slightly confident
    e. Not confident at all

—page break—

In these next set of questions, we will ask you to **rate the importance** of a series of factors. Then, on the next page, you will be asked to indicate **to what extent you experience** the same series of factors with the primary eczema healthcare provider you identified.

49. **How important are the following aspects of a visit to you** in order for you to discuss your/your child's experience and work together with your healthcare provider to identify

© 2022 Thibau IJ et al. *JAMA Dermatology.*

appropriate treatment options?
Please rate these factors from Not important at all to Absolutely essential. [Not important at all, Of little importance, Of Average importance, Very important, Absolutely essential]
Aspects about the visit

a. (TIME) Feeling like I am not rushed during my visit
b. (IN-PERSON VS. VIDEO) Having an in-person visit (as opposed to a virtual visit by video or phone)
c. (CONTINUATION OF CARE) Having follow-up about my/my child's treatment(s) or management plan (e.g., check-ins, discussions, additional visits)
d. (Tx ACCESS) Having follow-up about my/my child's treatment access (e.g., ability to purchase/obtain medication that was prescribed, insurance coverage)
e. (HCP ACCESS) Having the ability to access my/my child's healthcare provider outside the appointment (e.g., by phone, email, online portal)
f. (OFFICE ENVIRONMENT) Being in an office environment that makes me feel like I can engage in a discussion

Aspects about the provider

g. (TRUST) Having a healthcare provider that I can trust
h. (RACIAL SIMILARITY) Having a healthcare provider with the same race/ethnicity as me
i. (CULTURAL SENSITIVITY) Having a healthcare provider who expresses sensitivity to my culture
j. (GENDER SIMILARITY) Having a healthcare provider with the same gender as me
k. (PERCEIVED CLINICIAN RECEPTION) Having a healthcare provider value my input (based on my experience with eczema as a patient/caregiver)
l. (HEAR CONCERNS) Having a healthcare provider that listens to my perspectives about certain treatments (including concerns I might have)
m. (HOLISTIC CARE) Having a healthcare provider who asks about/is open to discussing my/my child's whole well-being (including mental health)
n. (GENUINE) Having a healthcare provider that expresses genuine concern about how I'm doing/my child is doing

Aspects about myself

o. (HEALTH LITERACY) Being able to understand the medical terms or concepts discussed during a visit
p. (COMMUNICATION SKILLS) Having the ability to articulate what I want to say about my/my child's condition (In other words, knowing how to express my/my child's symptoms, thoughts and feelings)
q. (SAFE SPACE) Having the comfort to articulate what I want to say about my/my child's condition (In other words, having a safe space to discuss all aspects of my/my child's condition)

© 2022 Thibau IJ et al. *JAMA Dermatology.*

Aspects about guidance

r. (SEEKING STRUCTURE) Having a handout/guide to structure the discussion
s. (GIVEN OPTION TO DISCUSS) Having my healthcare provider be the one to suggest we have a discussion about my/my child's treatment options (instead of having the healthcare provider immediately choose the treatment for me without my input)
t. (SEEKING DIRECTION) Having one clear recommendation from a healthcare provider about the treatment(s) I/my child should take
u. (SEEKING DIRECTION) Having an open-ended or multiple recommendations from a healthcare provider about the treatment(s) I/my child could take
v. (PREPARATION) Having information about treatment options beforehand

—page break—

50. Now, thinking about what you currently experience with the primary eczema healthcare provider you identified on previous pages, **to what extent are these aspects of a visit true for you?**

Please rate these factors from Never true to Always true.

[Never true, Rarely true, Sometimes true, Usually true, Always true]

Aspects about the visit

a. (TIME) I feel like I am not rushed during my visit
b. (IN-PERSON VS. VIDEO) I have in-person visits (as opposed to a virtual visit by video or phone)
c. (CONTINUATION OF CARE) I have follow-up about my/my child's treatment(s) or management plan (e.g., check-ins, discussions, additional visits)
d. (Tx ACCESS) I have follow-up about my/my child's treatment access (e.g., ability to purchase/obtain medication that was prescribed, insurance coverage)
e. (HCP ACCESS) I have the ability to access my/my child's healthcare provider outside the appointment (e.g., by phone, email, online portal)
f. (OFFICE ENVIRONMENT) The healthcare provider's office environment makes me feel like I can engage in a discussion

Aspects about the primary eczema healthcare provider identified earlier

g. (TRUST) I can trust my/my child's healthcare provider
h. (PERCEIVED CLINICIAN RECEPTION) The healthcare provider values my input (based on my experience with eczema as a patient/caregiver)
i. (HEAR CONCERNS) The healthcare provider listens to my perspectives about certain treatments (including concerns I might have)
j. (HOLISTIC CARE) The healthcare provider asks about/is open to discussing my/my child's whole well-being (including mental health)

© 2022 Thibau IJ et al. *JAMA Dermatology.*

k. (GENUINE) The healthcare provider expresses genuine concern about how I'm doing/my child is doing

Aspects about myself

l. (HEALTH LITERACY) I am able to understand the medical terms or concepts discussed during a visit
m. (COMMUNICATION SKILLS) I am able to articulate what I want to say about my/my child's condition (In other words, I know how to express my/my child's symptoms, thoughts and feelings)
n. (SAFE SPACE) I feel comfortable articulating what I want to say about my/my child's condition (In other words, I have a safe space to discuss all aspects of my/my child's condition)

Aspects about guidance

o. (SEEKING STRUCTURE) I have a handout/guide to structure the discussion
p. (GIVEN OPTION TO DISCUSS) The healthcare provider suggests we have a discussion about treatment options (instead of immediately choosing the treatment for me/my child without my input)
q. (SEEKING DIRECTION) I have one clear recommendation from the healthcare provider about the treatment(s) I/my child should take
r. (SEEKING DIRECTION) I have an open-ended or multiple recommendations from the healthcare provider about the treatment(s) I/my child could take
s. (PREPARATION) I have information about treatment options beforehand

—page break—

51. SOMETHING TO ADD Indicate to what extent you agree with the following statement: I feel that I have "something to add" to a discussion with a healthcare provider about my/my child's condition (in other words, my opinions as an eczema patient/caregiver have value).
    a. Strongly disagree
    b. Somewhat disagree
    c. Neither agree nor disagree
    d. Somewhat agree
    e. Strongly agree

52. MOTIVATION FOR SDM What would motivate you to engage in shared decision making with your/your child's healthcare provider? (write-in)

—page break—

© 2022 Thibau IJ et al. *JAMA Dermatology.*

53. TRUST/RAPPORT Have you ever sought a different healthcare provider out because you did not have a good relationship with your eczema healthcare provider?
    a. Yes
    b. No
54. TRUST/RAPPORT | IF YES: What was the reason you sought a different healthcare provider out? [write-in]

—page break—

55. INFO TO PREPARE Do you bring in your own information to an eczema care visit to prepare for your visit? These can include photos, notes, documentation, etc.
    a. Yes
    b. No
56. INFO TO PREPARE Where do you get information about **treatment options** to prepare for a visit? Select all that apply
    a. The National Eczema Association's website or print material
    b. The NIH or CDC website
    c. Some other association's website (e.g., American Academy of Dermatology, Asthma and Allergy Foundation of America)
    d. Some other health website (e.g., WebMD, Healthline, MayoClinic)
    e. Information shared in an online forum/support group
    f. Information from a social media platform (e.g., Facebook, Twitter, Instagram, LinkedIn)
    g. Information I got from my doctor or doctor's office (e.g., consultation, printouts, leaflets)
    h. A health app (please specify) ____
    i. Other (please specify) ____
    j. None: I don't get information on treatment options to prepare for a visit

—page break—

## Economic Factors

Finally, the following are some background questions. As mentioned earlier, all your answers will be kept strictly confidential and will be only used for general statistical analyses.

57. GEOGRAPHIC AREA In which U.S. state or territory is [your/the person with eczema's] household located?
    a. [Drop-down with 50 states, District of Columbia, Puerto Rico, U.S. Virgin Islands, Pacific Territories (including Guam, American Samoa, etc.)]
58. GEOGRAPHIC AREA What is your ZIP code?

© 2022 Thibau IJ et al. *JAMA Dermatology.*

a. _________

—page break—

The following are some background questions.  As mentioned earlier, all your answers will be kept strictly confidential and will be only used for general statistical analyses.

59. EMPLOYMENT What is your current employment status? (If more than one, select the category which best describes you).
    a. Employed full-time (30 hours or more)
    b. Employed part-time (less than 30 hours a week)
    c. Self-employed
    d. Out of work and looking for work
    e. Out of work but not currently looking for work
    f. Homemaker/caregiver
    g. Student
    h. Retired
    i. Disabled and unable to work
    j. Unable to work
    k. Other (specify _________ )
60. [IF D, E, OR J] Is your current status a result of COVID-19?
61. DISABILITY [IF I] Does your eczema keep you from working?
    a. Yes
    b. No

—page break—

The following are some background questions.  As mentioned earlier, all your answers will be kept strictly confidential and will be only used for general statistical analyses.

62. EDUCATION What is your highest level of education?
    a. Less than high school
    b. Completed some high school
    c. High school graduate
    d. Technical post-secondary
    e. Completed some college
    f. Four-year college degree
    g. Master's Degree/Doctorate
63. INCOME Which of the following categories best describes your 2020 household income before taxes?

© 2022 Thibau IJ et al. *JAMA Dermatology.*

a. $24,999 or less
b. $25,000 to $49,999
c. $50,000 to $74,999
d. $75,000 to $99,999
e. $100,000 to $124,999
f. $125,000 to $149,999
g. $150,000 or more

64. INSURANCE What best describes [your/the person with eczema's] type of medical insurance coverage?
    a. Employer-sponsored coverage
    b. Policy purchased on the commercial market
    c. Policy purchased on state/federal health exchange
    d. Medicare
    e. Medicaid or State assistance
    f. Tricare or VA benefit
    g. Unsure
    h. Do not have medical insurance

—page break—
SELECTING NEXT WILL END THE SURVEY AND BRING THE USER TO FORM ASSEMBLY DIRECTLY TO ENTER THEIR EMAIL ADDRESS

End of Survey — FormAssembly redirect

© 2022 Thibau IJ et al. *JAMA Dermatology.*
